# The role of ferroptosis in acute kidney injury: the preemptive mode of cell death and the bridging effect

**DOI:** 10.1080/0886022X.2025.2536732

**Published:** 2025-08-29

**Authors:** Huimeng Wang, Jiajia Sun, Yongsheng Luo, Xiaohu Li, Jinfeng Li

**Affiliations:** Department of Kidney Transplantation, The First Affiliated Hospital of Zhengzhou University, Zhengzhou, Henan, China

**Keywords:** AKI, ferroptosis, ferroptotic waves, immune cells, cell death

## Abstract

Ferroptosis represents a distinctive mechanism of cell death, differing from necroptosis, necrosis, and apoptosis. It is triggered by the accumulation of lipid peroxides, driven by iron-catalyzed reactions. This oxidative damage is essential for triggering the ferroptotic pathway. Compared with apoptosis and necroptosis, ferroptosis is activated earlier in acute kidney injury (AKI), serving as a preemptive mechanism of cell death. Ferroptosis acts as a link between synchronous waves of renal tubular cell death by triggering cell death amplification loops and connects cell damage with inflammatory responses, thus constituting a crucial stage in the progression of AKI. This paper discusses the mechanisms that trigger ferroptosis in AKI and how ferroptosis, as a preemptive mode of cell death, exacerbates AKI through ferroptotic waves, modulates inflammatory responses, triggering apoptosis, necroptosis, and pyroptosis.

## 1. Introduction

In 2012, Dixon defined ferroptosis as cell death triggered by the accumulation of lipid peroxides, driven by iron-catalyzed reactions. Ferroptosis is a finely regulated process that involves multiple molecular mechanisms. Ferroptosis is characterized by the intracellular iron retention, reduced glutathione (GSH) content, excessive production of reactive oxygen species (ROS), and increased levels of phospholipid polyunsaturated fatty acids (PUFAs) [1]. Ferroptosis exhibits distinct morphological, genetic, and mechanistic characteristics compared to previously discovered modes of regulated cell death, such as apoptosis, necroptosis, pyroptosis, and autophagy. Although research on ferroptosis is rapidly advancing, specific biomarkers of ferroptosis have not yet been conclusively identified.

**Figure 1.**
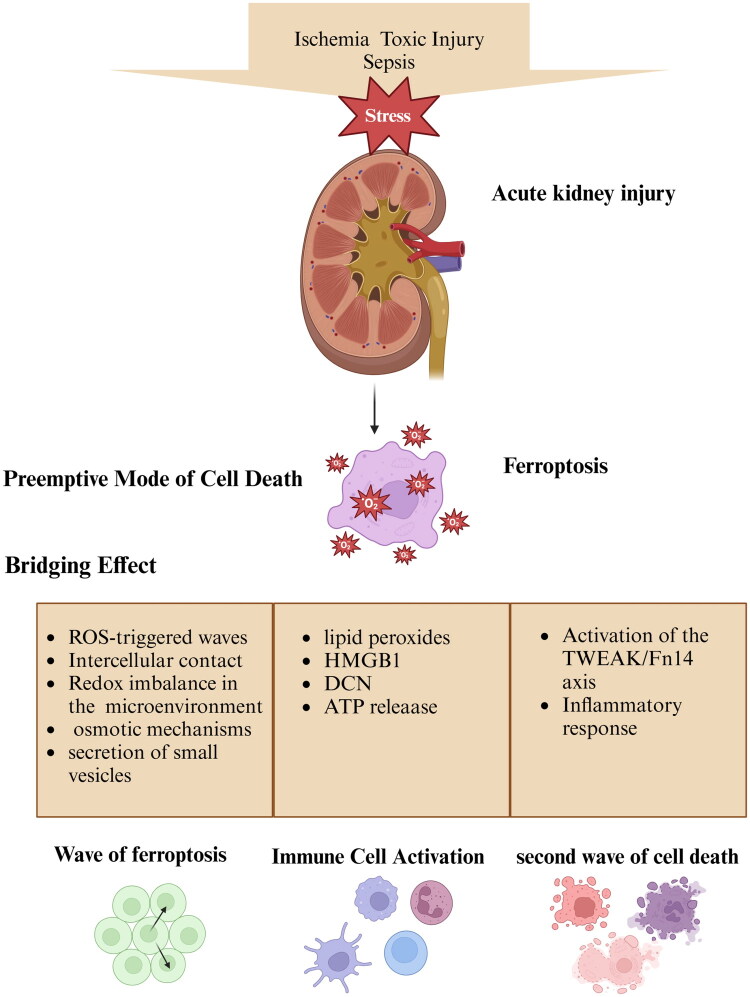
Ferroptosis acts as a pivotal death mechanism in acute kidney injury (AKI). In AKI, ferroptosis propagates in a wave-like manner through renal cells, leading to extensive nephron loss and synchronized cell death. Ferroptotic cells release lipid peroxidation products and damage-associated molecular patterns (DAMPs), which intensify crosstalk with immune cells. This interaction promotes immune cell activation and exacerbates renal inflammation. Additionally, ferroptosis can drive other forms of cell death through the triggering of death amplification loops, causing a secondary wave of cell death and further aggravating AKI. (HMGB1: High Mobility Group Box 1, DCN: decorin, ATP: Adenosine Triphosphate, TWEAK: Tumour Necrosis Factor (TNF)-Like Weak Inducer of Apoptosis, Fn14: Fibroblast Growth Factor-Inducible 14)

Single-cell sequencing analyses revealed that the ferroptosis pathway is highly active among renal tubular epithelial cells (RTECs), neutrophils and macrophages within the context of acute kidney injury (AKI) [2]. Furthermore, accumulating evidence has established the renoprotective effects of ferroptosis inhibitors in AKI [2]. Notably, recent studies utilizing the Art-Gd probe have achieved early AKI detection through Fe^2+^-targeted MRI imaging, identifying renal injury 24–48 h prior to creatinine level elevation [3]. These findings collectively underscore ferroptosis as a pivotal mechanism in both the initiation and progression of AKI. Thus, investigating the mechanism of ferroptosis in AKI and clarifying its impact on both the prognosis of AKI and its progression to chronic kidney disease (CKD) is significantly important (Figure 1).

**Figure 2.**
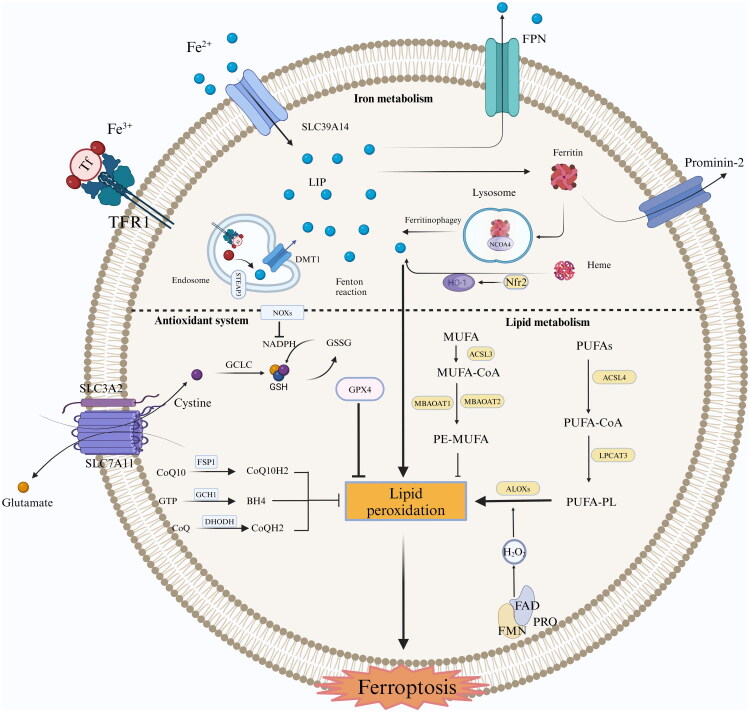
Mechanisms of ferroptosis. Ferroptosis is primarily induced by dysregulation in intracellular iron metabolism, lipid metabolism, and antioxidant systems. Further details can be found in the main text. (Ff: Transferrin, TFR1: Transferrin Receptor Protein 1, SLC39A14: Solute Carrier Family 39 Member 14, LIP: Labile Iron Pool, DMT1: Divalent Metal Transporter 1, STEAP3: Six-Transmembrane Epithelial Antigen of Prostate 3, FPN: Ferroportin, Nfr2: Nuclearrespiratoty factor 2, HO1: Heme Oxygenase-1, NOXs: Nitrogen Oxides, SLC3A2: Solute Carrier Family 3 Member 2, SLC7A11: Solute Carrier Family 7 Member 11, GCLC: Glutamate-Cysteine Ligase Catalytic Subunit, GPX4: Glutathione Peroxidase 4, FSP1: Ferroptosis-Suppressor-Protein 1, GTP: Guanosine Triphosphate, BH4: Tetrahydrobiopterin, DHODH: https://www.medchemexpress.cn/Targets/dihydroorotate-dehydrogenase.html Dihydroorotate Dehydrogenase, MUFA: Mono-Unsaturated Fatty Acid, ACSL3: Acyl-CoA Synthetase Long Chain Family Member 3, MBOAT1/2: Membrane-Bound O-Acyltransferase Domain-Containing 1, PL-MUFA: Phospholipid-Monounsaturated Fatty Acids, PUAFs: Polyunsaturated Fatty Acids, ACSL4: Acyl-CoA Synthetase Long Chain Family Member 4, LPCAT3: Lysophosphatidylcholine Acyltransferase 3, ALOXs: Arachidonic Acid Lipoxygenases, FAD: Flavin Adenine Dinucleotide, FMN: Flavin Mononucleotide, POR: Cytochrome P450 Oxidoreductase)

Ferroptosis, as an initial mode of cell death in AKI, not only causes considerable renal cell death, leading to the initiation of AKI, but may also act as an intermediary in AKI progression, thereby exerting a ‘bridging effect’ (Figure 2). The propagation of ferroptotic waves initiates the first synchronized wave of renal cell death, while simultaneously triggering apoptosis and necroptosis through the activation of their respective pathways, causing a second wave of renal cell death. Moreover, cells undergoing ferroptosis exhibit crosstalk with immune cells that leads to immune cell activation; such activated immune cells can in turn modulate renal cell damage and repair processes through various pathways. Therefore, ferroptosis may be an initiating factor of AKI and play a bridging role in modulating its progression.

## 2. The mechanism of ferroptosis

### 2.1. Iron metabolism and ferroptosis

When the intracellular iron-binding complex nears saturation, it contributes to the formation of a labile iron pool. Free divalent iron (Fe^2+^) generates ROS *via* the Fenton reaction. Overaccumulation of ROS can activate intracellular oxidative stress responses, leading to the destruction of proteins, nucleic acids, and lipids, ultimately triggering ferroptosis [4]. Thus, modulating intracellular free Fe^2+^ concentration can regulate cellular resistance to ferroptosis. Previous studies have established that the kidney plays a pivotal role in both iron filtration and reabsorption [5]. Notably, renal tissues exhibit particular susceptibility to iron dysregulation. During iron cycling, unstable ferrous iron (Fe^2+^) can participate in Fenton and Haber-Weiss reactions, undergoing redox cycling that ultimately promotes ferroptosis in renal cells [6]. Heat shock protein beta-1 can inhibit the expression of transferrin receptor(TFRC) [7], reducing iron uptake. Conversely, lactotransferrin and SLC39A14 can act as positive regulators of ferroptosis by facilitating iron intake [7,8]. Ferroportin-1 (FPN-1) and prominin-2 are capable of exporting iron ions and ferritin from the cell, respectively, thereby reducing intracellular iron load and enhancing resistance to ferroptosis [9,10]. The overexpression of FPN-1 inhibits celecoxib- and lapatinib-induced ferroptosis by promoting iron export, whereas the knockdown of FPN-1 increases ferroptosis [11]. Prominin-2 is abundantly expressed in the kidney and secreted in exosomes, which can be excreted into the urine [12]. Studies have shown that prominin-2 stimulates exosome-dependent iron export by forming ferritin-containing multivesicular bodies in epithelial cells, thus increasing resistance to ferroptosis [10]. However, the role of prominin-2 in AKI remains poorly understood, and further research is necessary to determine whether prominin-2-containing exosomes regulate ferroptosis in AKI.

Furthermore, ferroptosis can be modulated by manipulating the expression of genes associated with iron metabolism and their respective pathways. The silencing of iron-responsive element-binding protein 2 (IREB2) often results in the abnormal expression of genes associated with iron metabolism. Whereas, the upregulation of IREB2 enhances the synthesis of ferritin heavy chains and ferritin light chains, facilitating the formation of stable iron ions. Dysregulated expression of iron regulatory protein 1 can increase sensitivity to ferroptosis by mediating the expression of TFRC [1]. On the other hand, tristetraprolin is induced under conditions of cellular iron shortage and can reduce the synthesis of the iron-binding proteins by promoting the degradation of their mRNA, thus maintaining the cellular iron pool [13]. Nuclear factor erythroid 2-related factor 2 (NRF2) also plays a dual role in ferroptosis. On one hand, NRF2 exerts anti-ferroptotic effects by regulating antioxidant responses [14]. On the other hand, NRF2 enhances the transcription of HO-1 (Heme Oxygenase 1), which catalyzes heme degradation to release ferrous ions [15]. Ferroptosis caused by excessive HO-1 activity mediated by NRF2 is referred to as atypical ferroptosis [16]. Intracellular iron is stored mainly in the form of ferritin. Nuclear receptor coactivator 4-mediated ferritinophagy increases cellular concentrations of free divalent iron ions [8,17], thereby mediating autophagy-dependent ferroptosis.

### 2.2. Lipid metabolism and ferroptosis

During the initial phase of AKI, fatty acid oxidation is markedly upregulated [18]. In the ischemia-reperfusion injury (IRI)-induced AKI mouse model, significant increases in fatty acid signaling pathway activity, lipid levels, and fatty acid concentrations were observed at both 4 and 24-h post-reperfusion time points [19]. Similarly, persistent lipid droplet accumulation was detected in epithelial regions of the unilateral ureteral obstruction model [20]. Conversely, early intervention through lipid metabolism modulation was shown to effectively attenuate AKI [21]. The peroxidation of PUFAs constitutes a crucial step in promoting ferroptosis. Under oxidative stress conditions, the enhanced synthesis of PUFAs exacerbates intracellular lipid peroxidation. The distribution and abundance of PUFAs influence the extent of lipid peroxidation and the subsequent ferroptosis induction [22]. Free PUFAs are the important precursors for lipid peroxidation; however, they must be esterified into membrane phospholipids and oxidized to initiate ferroptosis. The synthesis of acyl-CoA derivatives from PUFAs is essential for this process. Enzymes that regulate the biosynthesis of membrane phospholipid PUFAs can either promote or inhibit ferroptosis [22]. Arachidonic acid (AA) and adrenic acid (AdA) are the primary substrates for lipid peroxidation [23]. ACSL4 and LPCAT3 participate in the reaction between free AA and adrenic acid with CoA, resulting in the formation of their respective AA-CoA and AdA-CoA derivatives [23–25]. This reaction promotes acid esterification into phospholipids, which is essential for ferroptosis. Subsequently, LPCAT3 facilitates the biosynthesis of these derivatives with membrane phosphatidylethanolamine (PE) to generate AA-PE and AdA-PE. Therefore, the absence of ACSL4 or LPCAT3 reduces the occurrence of ferroptosis. Studies have demonstrated that disrupting ACSL4 protein stability effectively inhibits ferroptosis in renal tubular epithelial cells, thereby ameliorating AKI [26]. Recently, the synthesis of alkyllysophospholipids, proposed to be an alternative source of PUFAs, was shown to be stimulated by peroxisome biogenesis factor 10 and peroxisome biogenesis factor 3, indicating the potential role of peroxisomes in promoting lethal lipid peroxidation linked to ferroptosis [27].

Lipid oxidases are mainly divided into three categories: lipoxygenases (LOXs), cyclooxygenase (PTGS2/COX2), and cytochrome P450 (CYP). Among them, LOXs are the most important enzymes for mediating ferroptotic cell death, as free PUFAs are their favored substrates for LOXs. It facilitates the peroxidation of PUFAs, thereby contributing significantly to the initiation and progression of ferroptosis. Thus, knocking out LOX can reduce the cell death mediated by ferroptosis. In addition, LOXs can catalyze the oxidation of phosphatidylethanolamine, thereby inducing ferroptosis [28]. However, LOXs are not the only lipid peroxidases capable of inducing ferroptosis. Research has shown that many cancer cells that can undergo ferroptosis exhibit low levels of ALOX enzyme expression [29]. In AKI models, the absence of Alox15 alone could not rescue AKI induced by GPX4 inactivation, indicating that lipid peroxidation can occur through a non-ALOX-dependent pathway and that other LOX isoforms may compensate for the lack of Alox15 [30]. Cytochrome P450 oxidoreductase, in combination with the cofactors flavin mononucleotide and flavin adenine dinucleotide, directly provides electrons to CYP enzymes, thereby promoting the peroxidation of PUFAs independently of ALOX [29]. It is currently unclear whether other oxygenases, such as epoxygenase and peroxygenase, have comparable roles in ferroptosis. PTGS2 is capable of oxidizing lysophospholipids, thereby mediating the occurrence of ferroptosis [31]. Additionally, PTGS2 could facilitate ferroptosis of neurons following brain injury [32]. However, although PTGS2 is overexpressed during ferroptosis, the inhibition of PTGS2 by indomethacin does not significantly inhibit ferroptosis [33]. Therefore, the precise mechanism of action of PTGS2 in ferroptosis remains unknown. Consequently, ongoing research is essential to elucidate the function of PTGS2 in AKI.

### 2.3. Antioxidant system and ferroptosis

Ferroptosis is regulated by multiple antioxidant systems, with the SLC7A11–GSH–GPX4 axis being the most critical [34–36]. GPX4, the key regulator of ferroptosis, reduces phospholipid hydroperoxides (PLOOH) to harmless phospholipid alcohols, thereby suppressing lipid peroxidation [37]. Previous studies have established that GPX4 depletion serves as a critical hallmark of ferroptosis in renal tubular epithelial cells during AKI [38]. Its activity depends on selenium and glutathione (GSH), which is synthesized *via* system xc⁻ (composed of SLC7A11 and SLC3A2) [39]. Modulation of SLC7A11 activity and expression influences ferroptosis susceptibility. The regulation of SLC7A11 activity and expression involves multiple factors, including cellular tumor antigen p53 (TP53) [40], Nrf2 [41], and BRCA1 associated protein 1 [42]. The inhibition of GPX4 serves as a vital downstream signal, with the overaccumulation of lipid peroxides following diminished GPX4 activity serves as a critical hallmark of ferroptosis [43]. However, GPX4-independent ferroptosis is still a viable pathway. For example, POR can participate in ML210 (a GPX4 inhibitor)-induced ferroptosis through mechanisms that operate independently of GPX4 [29]. TP53 can inhibit the expression of SLC7A11, but TP53-mediated ferroptosis does not necessitate GPX4 inhibition [29,44]. Studies have also shown that depleting GPX4 or SLC7A11 can decrease sensitivity to ferroptosis induced by Golgi stress [45]. In summary, despite the complexity of the mechanisms underlying ferroptosis, the SLC7A11–GSH–GPX4 axis is generally considered the most important intracellular anti-lipid peroxidation system.

Ferroptosis-suppressor-protein1 (FSP1), localized on the plasma membrane, generates reduced coenzyme Q10 (CoQH2) using NADPH, thereby neutralizing lipid peroxides independently of GPX4 [46]. Additionally, FSP1 can inhibit ferroptosis *via* membrane repair (ESCRT-III) or by regulating ubiquinol metabolism [47]. CD36-mediated FSP1 ubiquitination promotes phospholipid peroxidation, exacerbating ferroptosis in AKI [48]. The GCH1–BH4 pathway represents another GPX4-independent mechanism, where GCH1 synthesizes BH4 to selectively inhibit polyunsaturated fatty acid (PUFA) peroxidation [49,50]. BH4 deficiency increases ROS accumulation, while GCH1 upregulation enhances antioxidant defense [51]. Notably, GCH1 specifically protects against ferroptosis but not apoptosis [52]. These results indicate that GCH1 is potentially a selective target for ferroptosis. Mitochondrial dihydroorotate dehydrogenase (DHODH) cooperates with GPX4 to suppress ferroptosis by converting ubiquinone to CoQH2 [53–55], which scavenges lipid peroxides. DHODH upregulation compensates for GPX4 loss, highlighting its role in mitochondrial ferroptosis resistance [53]. Finally, the membrane-bound O-acyltransferase 1/2 (MBOAT1/2) –monounsaturated fatty acid (MUFA) system inhibits ferroptosis by remodeling phospholipids [56], increasing MUFA-containing phosphatidylethanolamines while reducing pro-ferroptotic PUFA-PLs [57]. Estrogen and androgen receptors upregulate MBOAT1/2, suggesting therapeutic potential [56].

## 3. The role of ferroptosis in AKI

### 3.1. Timing of cell death in AKI

The death of renal tubular cells is succeeded by processes of dedifferentiation, proliferation, and regeneration in the renal tubules, all accompanied by inflammatory responses [58]. Apoptosis was once considered the key factor in AKI, but recent research has identified other forms of cell death—including ferroptosis, necroptosis, and pyroptosis—as significant contributors to the pathophysiology of this condition [59]. Parenchymal cell necrosis appears to have a stronger association with the pathogenesis of AKI compared with other forms of cell death. Comprehensive intervention studies demonstrated that receptor-interacting protein kinase 3 (RIPK3) deficiency in mice can partially protect the kidneys from IRI-induced necroptosis [60,61], and the Nec-1 (a necroptosis inhibitor**)** phenotype recapitulates this protective effect. Similarly, sanglifehrin A (SfA) interferes with mitochondrial permeability transition (MPT)-regulated necroptosis (MPT-RN) to mildly protect against IRI as well [60,62]. However, dual therapy with Nec-1 and SfA does not completely restore creatinine levels or prevent kidney damage. After 40 min of ischemia and then reperfusion, all control mice died between 48 and 72 h, whereas mice treated with Fer-1 or 16–86 (a third-generation ferroptosis inhibitor) exhibited protection against functional and structural damage to the kidneys [62]. In addition, Fer-1 prevented morphological changes associated with synchronous tubular cell death *in vitro*. Erastin-treated tubular cells presented membrane protrusions, obvious deformation, and functional impairment, whereas those treated with ferroptosis inhibitors maintained normal morphology [62]. In another *in vitro* tubular toxicity experiment involving lactate dehydrogenase release, Fer-1 and the Nicotinamide adenine dinucleotide phosphate oxidase (Nox) inhibitor GKT protected against hydroxyquinoline iron-induced cell death, whereas Nec-1 did not have a protective effect [62]. Moreover, studies have indicated that in AKI, the expression of GPX4—a key regulator of ferroptosis—is significantly downregulated [63], accompanied by pronounced dysregulation of fatty acid metabolism and mitochondrial dysfunction [64]. With increasing research, various forms of cell death have been found to participate in AKI, but the precise sequence of these cell death modalities in AKI remains unclear.

In the different stages of AKI, the predominant modes of cell death may differ, and ferroptosis could be the preemptive mode of cell death in AKI, with ferroptosis-mediated inflammatory responses potentially driving other modes of cell death in this context. Previous research has indicated that ferroptosis is essential during the initial injury stages of AKI and that ferroptosis inhibitors can prevent morphological changes associated with synchronized renal tubular cell death, significantly improve renal function at 24 h. However, administering a Pan-caspase inhibitor (Z-VAD) or interfering with necroptosis (Nec-1, RIPK3-KO, or MLKL-KO) does not restore kidney early injury, as inhibiting necroptosis with Nec-1 or through RIPK3 knockout does not provide protective effects at early time points, despite the increases in RIPK3 and MLKL proteins in early stages of AKI [62,65]. Nevertheless, Nec-1, MLKL or RIPK3 deficiency has been shown to mitigate persistently elevated rates of cell death and renal dysfunction at 72–96 h [66]. MLKL phosphorylation is considered a marker of necroptosis pathway activation; in IRI-AKI models, Mlkl-KO mice show improved renal function at 48and 72 h post-reperfusion but show no significant improvement at early time points (6, 12, and 24 h) [67]. Studies also indicate that necroptosis may be activated in the later stages of death following reperfusion, as MLKL protein expression increases in a time-dependent manner, which is in agreement with necroptosis leading to the pathology of IRI [67]. Additionally, research has shown that ferroptosis mediates the activation of the TWEAK/Fn14 axis, resulting in elevated protein levels of MLKL and caspase-3, thereby causing apoptosis and necroptosis in AKI. Consistent with this, Fn14-KO preserves renal function at 72 h (but not 24 h) post-IRI-induced AKI in mice and reduces cell death levels [65], which coincides temporally with the protective effects of necroptosis intervention (Mlkl-KO). This temporal alignment further supports that the onset of necroptosis may be time-dependent, mainly contributing to tissue damage in the later stages of AKI. In summary, ferroptosis is more likely to be a preemptive mode of cell death in AKI, acting as a driver of renal inflammation and death propagation.

### 3.2. Bridging effect of ferroptosis

#### 3.2.1. Propagation of ferroptosis waves

**Figure 3.**
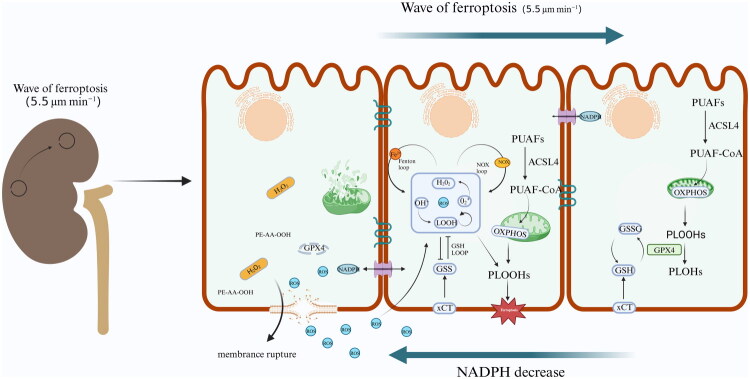
Wave-like propagation of ferroptosis between cells. Cells undergoing ferroptosis deplete their redox capacity, creating a NADPH gradient that propagates toward dying tubular cells. The excessive consumption of NADPH in these cells, relative to neighboring tubular cells, reduces their resistance to ferroptosis, facilitating its spread along the NADPH gradient. Additionally, ferroptotic cells release ROS, activating three ROS amplification loops: a GSH-mediated negative feedback loop, the Fenton reaction, and a NADPH oxidase (NOX)-mediated positive feedback loop, which together promote ferroptosis in adjacent cells. (H2O2: Hydrogen Peroxide, ROS: Reactive Oxygen Species, NADPH: Nicotinamide Adenine Dinucleotide Phosphate, GSH: Glutathione, GSSG: Oxidized Glutathione, OXPHOS: Oxidative phosphorylation, PLOOHs: Phospholipid Hydroperoxides, PLOHs: Phospholipid Hydroxides)

Ferroptosis is considered harmful to surrounding cells due to the release of toxic intracellular contents following cell death (Figure 3). However, other modes of cell death, including apoptosis, also generate waves of cell death, typically mediated by gap junction-mediated calcium transfer [68] or the release of potentially lethal signals such as TNF [69] that spread among cells, thereby causing excitotoxic necrotic waves. For example, the radiation-induced bystander effect, wherein cells neighboring those directly exposed to radiation show increased damage and death rates [70], may increase the frequency of death, but this spatial–temporal pattern of cell death is usually random. In contrast, ferroptosis propagates through cell populations with a wave-like appearance, exhibiting a highly nonrandom spatial–temporal pattern [62,70]. In many cases, the widespread propagation of the ferroptosis wave often leads to the almost complete disappearance of cell populations, significantly exacerbating IRI-induced kidney damage and delayed graft function during transplantation due to the synchronized renal tubule cell death [62]. Indeed, myocardial IRI often leads to the formation of extensive contiguous regions of necrotic cells, a phenomenon known as contraction band necrosis, presumably due to the spread of ferroptosis among cells [71,72]. Nevertheless, the molecular mechanisms underlying ferroptosis propagation are still not fully elucidated.

A recent series of compelling studies has identified the following factors as involved in the mechanisms of ferroptosis wave propagation: (i) ROS-triggered waves [73]; (ii) intercellular contact [74]; (iii) redox imbalance in the local microenvironment [75,76]; (iv) osmotic mechanisms independent of cell rupture [77]; and (v) secretion of small vesicles by ferroptotic cells [78,79]. As a signal of ferroptosis, ROS can act as trigger waves for the propagation of ferroptosis, priming cell populations to enter redox bistable state by activating ROS feedback loops. As a result, these redox bistable cells facilitate the propagation of ferroptosis through a sequence of ROS amplification–diffusion events, in which short-range ROS diffusion functions as a spatial coupling mechanism. Such trigger wave effectively overcomes the spatial constraints of simple diffusion, synchronizing ferroptosis across multiple cells and facilitating the emergence of tissue-wide cell death [73]. Another study demonstrated that the photoinducible degradation of the lipid reduction protein GPX4 can control the induction of ferroptosis with high temporal and spatial precision. The use of Opto-GPX4Deg to investigate cell death dynamics within cell groups revealed that ferroptosis spreads to adjacent cells in a distance-dependent manner, indicating a strong dependence on cell confluency for the propagation of ferroptosis and preferential effects on adjacent cells [74]. Furthermore, cells are connected by tubular compartments that share cytoplasm, which can particularly influence redox capacity. NADPH concentration gradients along renal tubules show inverse correlation with ferroptosis wave propagation direction, compromising antioxidant defenses against lipid peroxidation and thereby promoting wave spread [76,80]. Microenvironmental changes post-ferroptosis can also lead to the transmission of ferroptosis. Nishizawa demonstrated that the release of lipid peroxides from ruptured cells generates a lipid peroxidation gradient in the local microenvironment, which in turn induces the propagation of death signals [81]. Another study indicated that ferroptosis is an osmotic process in which the induction signal is propagated prior to cell rupture; this process, which involves cell swelling effects and is characterized by lipid peroxides and iron dependency, can be prevented by the addition of large osmolytes, the lipid peroxidation inhibitor liproxstatin-1, or deferoxamine [77]. Moreover, ferroptotic cells secrete a series of stimulatory signal transmitters that induce neighboring cells to undergo ferroptosis through various means. Small extracellular vesicles (sEVs) essential mediators in intercellular signaling, and their secretion by renal proximal tubular epithelial cells (RPTECs) is significantly upregulated during ischemia and hypoxia conditions. Hypoxic RTECs are secreting sEVs that possess unique ‘pro-injury’ characteristics and thereby trigger lipid peroxidation and the propagation of renal tubular ferroptosis waves [78]. Another study demonstrated the potential for sEVs secreted by kidney cells experiencing IRI to promote the spread of ferroptosis by delivering the lncRNA WAC-AS1. The sEVs secreted by renal cells under IRI reprogram glucose metabolism in neighboring RTECs by promoting Glutamine--Fructose-6-Phosphate Transaminase 1 expression and increasing hexosamine biosynthesis pathway flux, thereby increasing the addition of O-linked β-N-acetylglucosamine (O-GlcNAcylation). Significant inhibition of BACH2 O-GlcNAcylation in RTECs markedly suppresses ubiquitination-mediated degradation, and nuclear BACH2 inhibits the transcription of SLC7A11 and GPX4, reducing cellular oxidative defense mechanisms and thus facilitating the propagation of the ferroptosis wave [79]. Moreover, the transmission of ferroptotic waves has not only been confirmed in cellular models, but the possibility of wave-like propagation within the body also exists, potentially leading to extensive tissue damage. Ferroptosis is essential in the context of AKI and stroke or myocardial infarction, leading to the development of extensive areas of tissue necrosis. Intact zebrafish larvae undergo cell deformation after microperfusion with AA (a known driver of ferroptosis). These phenomena indicate that ferroptotic waves may potentially occurs *in vivo* and spread through tissues, leading to pathological necrosis and extensive tissue injury in various scenarios [82]. Chen and colleagues demonstrated that ferroptosis waves propagate at a constant speed (approximately 5.5 μm·min^−1^) over long distances (≥5 mm) in human cells [73]. Therefore, further revealing the mechanisms regulating ferroptosis and its propagation within cell populations will deepen our insights of AKI.

#### 3.2.2. Ferroptosis and inflammatory response

Following the onset of AKI, intercellular communication between parenchymal cells and immune cells is markedly enhanced. Natural killer T (NKT) cells and neutrophils serve as the initial responders at the site of injury during AKI, followed by monocytes, with dendritic cells (DCs) further activating lymphocyte immunity through antigen presentation. The roles of various immune cells, such as macrophages [83], DCs [84], T lymphocytes [85], and B lymphocytes [86] in AKI are increasingly being revealed. Much research has demonstrated that ferroptosis is highly active in diverse populations of renal tubular cells, along with immune cells under conditions of AKI [2,87,88]. As one of immunogenic cell death, ferroptosis can influence the phenotype and functionality of immune cells, thereby modulating the processes of injury and repair in AKI. Research indicates that genes related to ferroptosis play crucial roles in immunoregulation. The transcription factor nuclear factor-κB (NF-κB) is essential for immunoregulation and can be activated by TNF-α [89]. GPX4 can inhibit the activation of the TNF-α-mediated NF-κB signaling pathway and mitigate necrotic inflammation [90,91]. ACSL4-dependent lipid biosynthesis is an essential step in the process of ferroptosis [92]. Additionally, ACSL4 is implicated in inflammatory responses, and its expression is positively associated with the abundance of immune cells [93].

Ferroptosis, a highly immunogenic form of immunogenic cell death, critically contributes to renal cell death amidst the backdrop of AKI. It activates inflammatory response through the release of damage-associated molecular patterns (DAMPs) and inflammatory mediators [94]. While undergoing ferroptosis, cells promote the production of DCs, which, as they mature and activate, demonstrate an increased phagocytic capacity [95]. Compared with live cells, dying cells are more efficiently phagocytosed by DCs. Subsequently, DCs mature and express the CD86 and MHCII and release higher levels of IL-6 [96]. Research has shown that cells experiencing ferroptosis release DAMPs, such as ATP and HMGB1. These DAMPs promote the maturation of DCs and activate adaptive immunity mediated by cytotoxic T cells. T-cell-derived IFN-γ promotes the binding of signal transducer and activator of transcription 1 to the transcription start site of SLC7A11, thereby enhancing its transcription and ultimately leading to ferroptosis [97]. Lipid peroxides, oxidized phospholipids (oxPLs), and lysophospholipids are released directly from ferroptotic cells. These peroxides are a unique category of DAMPs released during ferroptosis that have pronounced mutagenic effects that impact immunity [98]. Conversely, evidence suggests that phospholipid oxidation products generated by ALOX12/15 inhibit the maturation of DCs and fine-tune the differentiation of Th17 cells [98]. Oxidized lipid-containing liposomes are taken up by DCs, inhibit antigen cross-presentation, and subsequently silence the CD8+ T-cell response [99]. Another study revealed that after cardiac transplantation, ferroptosis is essential in the injured myocardium and coordinates neutrophil recruitment to the injured site through the TLR4/TRIF pathway and type I interferon signaling [71]. Subsequently, the activation of immune cells can, in turn, regulate the activity of parenchymal cells. Historically, it was believed that the primary function of renal DCs was antigen presentation to T cells. However, DCs can release TNF-α, which acts as a principal inflammatory mediator during the initial 24 h following IRI. TNF-α binds to renal endothelial cells and induces cell death [100], undoubtedly exacerbating AKI.

**Figure 4.**
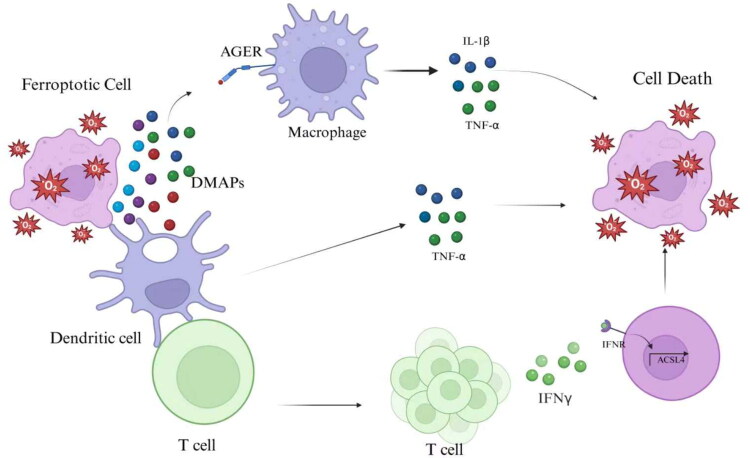
Crosstalk between ferroptotic cells and immune cells. DAMPs released by ferroptotic cells activate macrophages and dendritic cells. Activated dendritic cells promote the secretion of interferon-gamma (IFN-γ) by activating T cells. IFN-γ enhances ACSL4 transcription, further promoting ferroptosis in parenchymal cells. DAMPs released by ferroptotic cells bind to AGER/RAGE receptors, activating the NF-κB pathway, which triggers an inflammatory response in macrophages. Activated macrophages, in turn, modulate the sensitivity of parenchymal cells to ferroptosis through the production of TNF-α and IL-1β. (DAMPs: Damage-Associated Molecular Patterns, AGER: Advanced Glycosylation End-Product Specific Receptor, IL-1β: Interleukin-1beta, TNF-α: Tumour Necrosis Factor-alpha, IFN-γ: Interferon gamma)

Many investigations have demonstrated that the crosstalk between renal cells and macrophages is vital for controlling the progression of AKI and the associated repair mechanisms [101]. Single-cell sequencing results further confirm that macrophages are the most active immune cell populations. They serve as primary senders, receivers, and modulators of ligand-receptor signaling among parenchymal cells [2]. As a significant participant in AKI, ferroptosis reprograms the phenotype and function of macrophages, thereby playing a vital role in AKI and its associated injury and repair processes [102,103]. The functional state of macrophages not only affects renal injury and repair [104] but also influences parenchymal cell resistance to ferroptosis [105]. Products resulting from parenchymal cell ferroptosis can induce macrophage polarization. HMGB1, a DAMPs released from ferroptotic cell, activates inflammatory responses [91]. Additionally, decorin, a novel DAMPs released by cells undergoing ferroptotic cell death, may function as a distinctive biomarker for ferroptosis [106]. Both HMGB1 and decorin can activate receptor for advanced glycosylation end products (AGER/RAGE). RAGE activation subsequently triggers the NF-κB pathway, causing inflammation and increasing the production and release of proinflammatory cytokines, including IL-6 and TNF-α [91,106]. Another study revealed that renal cells undergoing ferroptosis release spliceosome-associated protein 130, which promotes M1 polarization *via* Mincle signaling, exacerbating renal damage [107]. Furthermore, ferroptosis can modulate macrophage phenotype through alterations in cholesterol metabolism and fatty acid oxidation, leading to the generation of distinct functional phenotypes [88,108]. The functional phenotypes of macrophages can, in turn, affect parenchymal cell resistance to ferroptosis. Previous studies suggested that M1 macrophages can increase mitochondrial damage and ROS accumulation in RTECs, thereby promoting ferroptosis [107]. The release of TNF-α by M1 macrophages destabilizes GPX4, thereby decreasing cellular resistance to ferroptosis [58]. Additionally, TNF-α upregulates ACSL3, which encodes an essential enzyme involved in acyl-CoA synthesis, suggesting that TNF-α facilitates intracellular lipid accumulation and thus creates a microenvironment conductive to inflammation and ferroptosis [109]. Additionally, the M1 macrophage marker IL-1β has been linked to renal lipid homeostasis. IL-1β induces human glomerular mesangial cells to upregulate LOX-1 expression and take up more oxidized low-density lipoprotein [110,111]. Consequently, excessive IL-1β-induced lipid accumulation may trigger renal cell ferroptosis. Studies have shown that M1 macrophage-produced IL-6 can control the transcription of the short peptide hormone hepcidin through a single mechanism. Hepcidin binds to FPN, resulting in hepcidin-FPN complex internalization and rapid degradation in lysosomes [112]. Increased hepcidin levels significantly reduces FPN expression, exacerbating ferroptosis. In contrast, M2 macrophages play an immunosuppressive role and promote tissue repair in AKI [113]. Recent studies reported that M2 macrophages can increase cancer cell resistance to ferroptosis. M2 macrophages enhance tumor cell resistance to ferroptosis by secreting taurocholic acid to activate the liver X receptor-α and sterol regulatory element-binding protein-1 pathway, which inhibits ferroptosis [105]. Currently, research on immune cells and ferroptosis in AKI primarily focuses on the direct regulatory effects of immune cells on ferroptosis. There is still insufficient research regarding the interaction between ferroptotic cells and immune cells (Figure 4). Studying how immune cells regulate the timing of parenchymal cell death may reveal the sequence of such death during AKI and clarify the mechanisms behind the mass death of these cells following injury.

#### 3.2.3. Ferroptosis drives diverse cell death mechanisms in AKI

Ferroptosis is initiated early during AKI and may consequently trigger various downstream effects. Research has shown that ferroptosis can activate inflammatory responses, which in turn mediate the death of RTECs [114]. Another study further demonstrated that ferroptosis can activate inflammation, subsequently inducing a second wave of cell death through the activation of the TWEAK/Fn14 axis during AKI [115]. Studies indicate that Fn14-KO mice retain better kidney function and exhibit decreased levels of cell death at 72 h but not at 42 h after IRI, with this protection overlapping the effects of necroptosis inhibition (e.g., in MLKL-KO mice). Furthermore, the absence of Fn14 reduces the levels of necrotic proteins such as RIPK1, MLKL, RIPK3, and caspase-3, indicating that ferroptosis can partially regulate the occurrence of apoptosis and necroptosis by activating the TWEAK/Fn14 axis [66]. Another study indicated that necroptosis may be activated in the later stages after reperfusion, as evidenced by the time-dependent increase in MLKL protein levels, which aligns with the pathology of IRI-induced necroptosis [67]. *In vitro* studies have also shown that TWEAK activates apoptosis in the presence of proinflammatory cytokines but activates necroptosis in the presence of caspase inhibitors, whereas both apoptosis and necroptosis rely on Fn14 activation [116]. Additionally, ATP—a well-characterized DAMP in immunogenic cell death—is passively released during ferroptosis, specifically acting as an immunogenic signal in early ferroptosis [117] that can trigger the assembly of inflammasomes, the recruitment and activation of caspase-1, and ultimately induce pyroptosis [118]. Therefore, Ferroptosis may occur early in AKI, death signals are released that trigger subsequent waves of apoptosis, necroptosis, and pyroptosis. Ferroptosis-mediated damage has also been observed in other organs. Severe intestinal injury resulting from ischemia/reperfusion can lead to acute injury to distant organs, such as the liver and lungs, that is likely mediated by ferroptosis, as ferroptosis inhibitors can improve both intestinal IRI and distant organ damage caused by intestinal injury [60,119]. Thus, ferroptosis may trigger the initial wave of cell death; then, ferroptotic cells release intracellular organelles and DAMPs, thereby enhancing inflammatory responses and recruiting immune cells [120]. The recruitment of pro-inflammatory factors may initiate a secondary wave of renal cell death, driven by cytokine-induced mechanisms and immune cell-mediated effects. Despite the significance of ferroptosis in AKI, the mechanism of renal tubular cell death in AKI may be complex and variable. Inhibiting a single mode of death may increase the sensitivity of cells to another type of cell death. When cell death is inevitable, cells may engage in various modes of death depending on the specific context. Studies demonstrate a connection between ferroptosis and necroptosis, in that renal tubular epithelial cells exposed to ferroptosis inhibitors become more sensitive to necroptosis. As previously mentioned, a combined inhibitor of apoptosis or necroptosis can increase the efficacy of ferroptosis inhibitors [67]. Therefore, a further understanding of the different types of cell death at different stages of AKI, as well as the interplay among these modes, is essential. Clarifying the timing of different forms of cell death based on AKI stages will enable more targeted treatment approaches for acute kidney injury.

## 4. Treatment of AKI by targeting ferroptosis

The pathogenesis of AKI is intricate, and ferroptosis is identified as a crucial mediator of renal damage [121]. Numerous studies have demonstrated that ferroptosis serves as a precise therapeutic target for AKI, and its inhibition can significantly attenuate disease progression. At present, ferroptosis inhibitors include antioxidants, iron homeostasis regulators, lipid peroxidation pathway inhibitors, lipid synthesis pathway regulators, nitrogen oxides and selenium supplements, impacting different aspects of ferroptosis. Several ferroptosis inhibitors (Ferrostatin-1, 16–86, and Liproxstatin-1) have been shown to ameliorate AKI [122–124]. Ferrostatin-1 can inhibit ferroptosis by regulating GSH/GPX4 signaling. Moreover, ferrostatin-1 can diminish lipid peroxides and inhibit cell death by reducing ROS levels in various disease models, including AKI models [125]. In a severe IRI model, mice treated with 16–86 showed greater protection from functional and structural organ damage [62]. Liproxstatin-1 inhibits ferroptotic cell death by delaying the overaccumulation of lipid hydroperoxides through increasing GSH and restoring GPX4 levels [126]. Liproxstatin-1 alleviates AKI by inhibiting ferroptosis [127] and significantly reducing renal lipid peroxidation and renal histopathological damage in AKI rats [30,128]. In addition, antioxidants including vitamin E and curcumin have been demonstrated to contribute to acute injury by scavenging peroxy radicals, inhibiting the formation of phospholipid hydroperoxides, and inhibiting ferroptosis [129,130]. The iron regulators can chelate iron ions in cells, thus alleviating the occurrence of ferroptosis. Kidney-scavenging quantum dot–drug conjugates composed of carbon quantum dots, desferoxamin and polyethylene glycol can inhibit ferroptosis and reduce the occurrence of AKI by scavenging the ROS produced by the Fenton reaction [131]. Beyond conventional ferroptosis inhibitors, several other compounds have been identified as having inhibitory effects on ferroptosis. Recent research demonstrated that polydatin, a natural polyphenol, has strong biological activity and unique potential in regulating iron metabolism. Polydatin can significantly inhibit the accumulation of excessive Fe2+ and ROS in AKI to alleviate the progression of AKI [132]. Drugs that inhibit the biological activity of LOX and ACSL4 (such as zileuton and thiazolidinedione) can also prevent ferroptosis in AKI by inhibiting lipid peroxidation [124,133]. In addition, selenium supplementation can effectively inhibit ferroptosis in AKI by increasing GPX4 activity and reducing lipid peroxide accumulation. Se/albumin nanoparticles can inhibit ferroptosis in RTECs during AKI. Vitamin D receptor activation attenuates AKI by targeting GPX4 to inhibit tubular epithelial cell ferroptosis [134]. Emerging studies have highlighted the considerable promise of ferroptosis-targeting nanomaterials for AKI intervention. Specifically, the Fe@Ba nanozyme was synthesized through coordination-driven self-assembly of Fe³^+^ ions with bioactive antioxidant baicalein ligands. This novel nanozyme potently inhibits ferroptosis in AKI [135].

However, although many drugs have been proven effective in animal models and cell experiments, their clinical application is limited by poor metabolic stability in plasma and systemic side effects (6). With increasing understanding of the mechanisms of ferroptosis, inhibiting ferroptosis could represent a more targeted treatment approach for AKI.

## 5. Conclusions

Ferroptosis is crucial in AKI, and many experiments have demonstrated that targeting the ferroptosis pathway can protect renal function. As research has progressed, numerous regulatory factors associated with ferroptosis have been confirmed. Ferroptosis drives AKI progression through downstream effects. Ferroptosis can propagate in a wave-like manner within renal tubular cells, leading to synchronous necrosis and ultimately promoting the progression of AKI. Furthermore, ferroptosis can exacerbate renal inflammation by reshaping the phenotype of renal macrophages and recruiting immune cells. This inflammatory cascade triggers secondary cell death (apoptosis, necroptosis, and pyroptosis) in adjacent tubules, forming a self-amplifying loop that worsens AKI. However, in the realm of ferroptosis research, many unknowns still exist, and the timing of cell death modalities in AKI remains unclear. The types of cell death at different stages of AKI and the connections between various modes of cell death require further clarification. Moreover, the molecular and comprehensible mechanisms of ferroptosis related to AKI are still poorly understood, and specific biomarkers for ferroptosis have not yet been identified. Current understanding of ferroptosis in AKI is largely derived from animal and cellular studies, while clinical evidence from human subjects remains limited. Therefore, it is particularly important to validate ferroptosis mechanisms in human-based studies. The integration of fundamental research with clinical applications may provide novel insights for advancing AKI treatment. Therefore, further exploration of ferroptosis in the context of AKI holds significant potential. With a growing recognition of the ferroptotic regulatory mechanisms, promising novel therapeutic approaches for AKI are likely to arise.

## Data Availability

All data relevant to this review are included in the text, references, tables and figures.
